# Functional connectivity density alterations in schizophrenia

**DOI:** 10.3389/fnbeh.2014.00404

**Published:** 2014-11-19

**Authors:** Chuanjun Zhuo, Jiajia Zhu, Wen Qin, Hongru Qu, Xiaolei Ma, Hongjun Tian, Qingying Xu, Chunshui Yu

**Affiliations:** ^1^Department of Radiology and Tianjin Key Laboratory of Functional Imaging, Tianjin Medical University General HospitalTianjin, China; ^2^Functional Neuroimaging Laboratory, Department of Psychiatry, Tianjin Mental Health Center, Tianjin Anding HospitalTianjin, China; ^3^Tianjin Anning HospitalTianjin, China

**Keywords:** schizophrenia, resting state, functional magnetic resonance imaging, functional connectivity density, functional connectivity strength

## Abstract

**Background:** Schizophrenia is characterized by altered resting-state functional connectivity. Most previous studies have focused on changes in connectivity strengths; however, the alterations in connectivity density in schizophrenia remain largely unknown. Here, we aimed to investigate changes in resting-state functional connectivity density (rsFCD) in schizophrenia.

**Methods:** A total of 95 schizophrenia patients and 93 sex- and age-matched healthy controls (HCs) underwent resting-state functional MRI examinations. The rsFCD, which reflects the total number of functional connections between a given brain voxel and all other voxels in the entire brain, was calculated for each voxel of each subject. Voxel-based comparisons were performed to identify brain regions with significant rsFCD differences between patients and controls (*P* < 0.05, corrected).

**Results:** Compared with HCs, patients with schizophrenia showed significantly increased rsFCD in the bilateral striatum and hippocampus and significantly decreased rsFCD in the bilateral sensorimotor cortices and right occipital cortex. However, the rsFCD values of these brain regions were not correlated with antipsychotic dosage, illness duration, or clinical symptom severity.

**Conclusions:** The striatal and hippocampal regions and parietal-occipital regions exhibited completely different changes in rsFCD in schizophrenia, which roughly correspond to dopamine activity in these regions in schizophrenia. These findings support the connectivity disorder hypothesis of schizophrenia and increase our understanding of the neural mechanisms of schizophrenia.

## Introduction

Schizophrenia is a devastating psychiatric disease that is characterized by hallucinations, delusions, loss of initiative, and cognitive deficits (van Os and Kapur, 2009; Gustavsson et al., 2011). Although there is no accepted theory accounting for schizophrenia (Rubinov and Bullmore, 2013), connectivity disturbance has been considered a hallmark of schizophrenia. Using diffusion tensor imaging (DTI), extensive anatomical connection impairments have been found in schizophrenia (van den Heuvel et al., 2010, 2013; Wang et al., 2012). Using functional magnetic resonance imaging (fMRI), resting-state functional connectivity strength (rsFCS) has been proposed to explore intrinsic association between two brain regions. It measures temporal correlations of spontaneous fluctuations in brain activity between different pairs of brain areas (Biswal et al., 1995). rsFCS may reflect intrinsic functional organization of the brain. Schizophrenia patients have shown rsFCS alterations in multiple specific brain regions and networks (Karbasforoushan and Woodward, 2012; Woodward et al., 2012; Moran et al., 2013; Orliac et al., 2013; Shinn et al., 2013; Argyelan et al., 2014; Klingner et al., 2014; Kraguljac et al., 2014; Liu et al., 2014). For example, schizophrenia patients have decreased inter-regional rsFCS between the amygdala and frontal lobe (Liu et al., 2014) and between the hippocampus and parietal lobe (Kraguljac et al., 2014). They also exhibit decreased intra-network rsFCS in the default-mode network (DMN) and salience network (SN) (Orliac et al., 2013). The patients also have decreased inter-network rsFCS between the SN and central executive network (CEN) and DMN (Moran et al., 2013). Both the decreased (Woodward et al., 2012) and increased (Klingner et al., 2014) prefrontal-thalamic rsFCS have been reported in schizophrenia, although the increased somatosensory-thalamic rsFCS is rather consistent (Woodward et al., 2012; Klingner et al., 2014). However, most previous studies investigating rsFCS abnormalities in schizophrenia used hypothesis-driven methods based on a priori selection of a region of interest (ROI) (Woodward et al., 2012; Shinn et al., 2013; Klingner et al., 2014; Kraguljac et al., 2014; Liu et al., 2014), which cannot provide a full picture of the brain rsFCS changes in schizophrenia. Thus, several studies have investigated rsFCS alterations across the entire brain in schizophrenia using a multiple ROI-based method (Liang et al., 2006; Venkataraman et al., 2012). Liang et al. found decreased rsFCS between many pairs of brain regions in schizophrenia (Liang et al., 2006). However, Venkataraman et al. reported that patients with schizophrenia exhibited co-existing patterns of increased rsFCS between parietal and frontal regions, and decreased rsFCS between parietal and temporal regions and between the temporal cortices bilaterally (Venkataraman et al., 2012). Almost all previous studies have focused on rsFCS changes in schizophrenia; however, connection number changes of each voxel across the entire brain in schizophrenia are still unclear.

Recently, resting-state functional connectivity density (rsFCD) has been developed to measure the number of resting-state functional connections of a given voxel with all other voxels in the entire brain (Tomasi and Volkow, 2010, 2011a,b). rsFCD mapping is an ultra-fast voxel-wise data-driven method for computing rsFCD with high spatial resolution (3-mm isotropic) (Tomasi and Volkow, 2010, 2011a,b) and has been used to identify age-related connectivity changes, connectivity differences across genetic backgrounds, and connectivity alterations in some disorders (Tomasi and Volkow, 2012a,b, 2014; Tian et al., 2013). rsFCS measures connectivity strength between two voxels or regions or networks, which reflects a one-to-one relationship. However, rsFCD reflects a one-to-many relationship. A greater rsFCD value for a particular voxel indicates that this voxel is functionally connected to a greater number of other brain voxels and suggests that this voxel plays a more important role in the information processing of the brain than those voxels with lower rsFCD values. Thus, rsFCS and rsFCD reflect different connectivity properties. Investigating rsFCD changes in schizophrenia may provide extra information (change in importance of a voxel in information processing) that cannot be obtained from an rsFCS analysis. This information may facilitate our understanding of the neural mechanisms of schizophrenia from a new perspective.

In the present study, we compared rsFCD differences between 95 schizophrenia patients and 93 healthy controls (HCs) to identify brain regions with altered rsFCD in schizophrenia.

## Materials and methods

### Subjects

A total of 200 right-handed individuals were enrolled in the present study, including 98 schizophrenia patients and 102 HCs. The diagnoses and illness durations of schizophrenia were determined by the consensus of two experienced clinical psychiatrists using the Structured Interview for DSM-IV Axis I Disorders. All HCs were screened using the non-patient edition of the SCID to confirm a lifetime absence of psychiatric illnesses. In addition, all of the HCs were interviewed to exclude individuals with a known history of psychiatric illness in first-degree relatives. Exclusion criteria for all subjects were a history of head trauma with consciousness disturbances lasting more than 5 min, a history of drug or alcohol abuse, pregnancy, and any physical illness such as cardiovascular disease or neurological disorders, as diagnosed by an interview and medical records review. A professional radiologist assessed image quality slice-by-slice, and three patients and 9 HCs with poor image quality were excluded. Consequently, 95 schizophrenia patients and 93 HCs were included in the statistical analysis. Clinical symptoms of psychosis were quantified using the Positive and Negative Syndrome Scale (PANSS). The antipsychotic dosages are reported (Table 1) as the chlorpromazine equivalents calculated based on clinically equivalent dosing estimates (Gardner et al., 2010). For each schizophrenia patient, the chlorpromazine equivalent was estimated according to the antipsychotic drugs and dosages used in the latest week before the MRI scan. The Medical Research Ethics Committee of Tianjin Medical University General Hospital approved this study. After a complete description of the study, written informed consent was obtained from each subject.

**Table 1:** **Demographic and clinical characteristics**.

**Characteristics**	**Schizophrenia patients**	**Comparison subjects**	***P*-value**
Number of subjects	95	93	
Age (years)	33.6 (7.8)	33.0 (10.2)	0.633
Sex (female/male)	41/54	48/45	0.246
Antipsychotic dosage (mg/d) (chlorpromazine equivalents)	446.5 (341.6)		
Duration of illness (months)	121.4 (92.8)		
PANSS			
Positive score	17.1 (7.9)	–	
Negative score	20.3 (9.1)	–	
General score	34.1 (10.8)	–	
Total score	71.5 (23.2)	–	

*Data are shown as the means (SD). Abbreviations: PANSS, The Positive and Negative Syndrome Scale*.

### MRI data acquisition

MRI data were acquired using a 3.0-Tesla MR system (Discovery MR750, General Electric, Milwaukee, WI, USA). Tight but comfortable foam padding was used to minimize head motion, and earplugs were used to reduce scanner noise. Sagittal 3D T1-weighted images were acquired using a brain volume sequence with the following parameters: repetition time (*TR*) = 8.2 ms; echo time (*TE*) = 3.2 ms; inversion time (*TI*) = 450 ms; flip angle (*FA*) = 12°; field of view (*FOV*) = 256 × 256 mm; matrix = 256 × 256; slice thickness = 1 mm, no gap; and 188 sagittal slices. Resting-state fMRI data were acquired using a gradient-echo single-short echo planar imaging sequence with the following parameters: *TR*/*TE* = 2000/45 ms; *FOV* = 220 × 220 mm; matrix = 64 × 64; *FA* = 90°; slice thickness = 4 mm; gap = 0.5 mm; 32 interleaved transverse slices; and 180 volumes. All subjects were instructed to keep their eyes closed, relax, move as little as possible, think of nothing in particular, and not fall asleep during the fMRI scans.

### fMRI data preprocessing

Resting-state fMRI data were preprocessed using SPM8 (http://www.fil.ion.ucl.ac.uk/spm). The first 10 volumes for each subject were discarded to allow the signal to reach equilibrium and the participants to adapt to the scanning noise. The remaining volumes were corrected for the acquisition time delay between slices. Then, realignment was performed to correct the motion between time points. All subjects' fMRI data were within the defined motion thresholds (i.e., translational or rotational motion parameters less than 2 mm or 2°). We also calculated frame-wise displacement (FD), which indexes the volume-to-volume changes in head position. There were no significant group differences in FD (*t* = 0.56, *P* = 0.58) between patients (0.117 ± 0.007) and controls (0.113 ± 0.006). Several nuisance covariates (six motion parameters, their first time derivations, and average BOLD signals of the ventricular and white matter) were regressed out from the data. Recent studies have reported that the signal spike caused by head motion significantly contaminated the final resting-state fMRI results even after regressing out the linear motion parameters (Power et al., 2012). Therefore, we further regressed out spike volumes when the FD of the specific volume exceeded 0.5. The datasets were then band-pass filtered in a frequency range of 0.01–0.08 Hz. In the normalization step, individual structural images were linearly co-registered with the mean functional image; the structural images were then linearly co-registered to MNI space. Finally, each filtered functional volume was spatially normalized to MNI space using co-registration parameters and resampled into a 3-mm cubic voxel.

### rsFCD calculation

The rsFCD of each voxel was calculated using an in-house script that was written in the Linux platform according to the method described by Tomasi and Volkow (2010, 2011a,b). Pearson's linear correlation evaluated the strength of the functional connectivity between voxels. Two voxels with a correlation coefficient of *R* > 0.6 were considered significantly connected. This threshold was proposed to be the optimal threshold for calculating rsFCD in a previous study (Tomasi and Volkow, 2010). The rsFCD calculation was restricted to a cerebral gray matter (GM) mask. The rsFCD at a given voxel x_0_ was computed as the total number of functional connections, k(x_0_), between x_0_ and all other voxels. This calculation was repeated for all x_0_ voxels in the brain. The grand mean scaling of rsFCD was obtained by dividing by the mean value of all brain voxels to increase the normality of the distribution. Finally, the rsFCD maps were spatially smoothed using a 6 × 6 × 6 mm full-width at half maximum (FWHM) Gaussian kernel. To explore the effects of the selection of correlation thresholds on our rsFCD analysis, rsFCD maps were also calculated based on two additional correlation thresholds (*R* > 0.2 and 0.4).

### Gray matter volume calculation

The gray matter volume (GMV) of each voxel was calculated using voxel-based morphometry (VBM), as implemented in the VBM8 toolbox (http://dbm.neuro.uni-jena.de/vbm.html). Structural MR images were segmented into GM, white matter and cerebrospinal fluid using the standard segmentation model. After an initial affine registration of the GM concentration map into Montreal Neurological Institute (MNI) space, GM concentration images were nonlinearly warped using diffeomorphic anatomical registration through the exponentiated Lie algebra (DARTEL) technique, and the results were resampled to a voxel size of 3 × 3 × 3 mm. The relative GMV of each voxel was obtained by multiplying the GM concentration map by the non-linear determinants that were derived from the spatial normalization step. Finally, the GMV images were smoothed using a Gaussian kernel of 6 × 6 × 6 mm FWHM. After spatial preprocessing, the smoothed GMV maps were used for statistical analyses.

### Statistical analysis

Group differences in rsFCD were compared in a voxel-wise manner using a general linear model with age and sex as nuisance variables. A permutation-based inference tool for nonparametric statistics in FMRIB's diffusion toolbox (FSL 4.0, http://www.fmrib.ox.ac.uk/fsl) was used to perform this analysis. The number of permutations was set to 5000, and the significance threshold was set at *P* < 0.05 after correcting for family-wise error (FWE) using the threshold-free cluster enhancement (TFCE) option in FSL. To exclude the possible effects of GMV on rsFCD changes, we repeated the group comparisons with the GMV as an additional covariate of no interest at the voxel-wise level. Moreover, the same statistical steps were applied to rsFCD at the thresholds of *R* > 0.2 and 0.4.

The mean rsFCD of each cluster with significant group differences was extracted for each subject. The partial correlation coefficient was used to test the association between rsFCD and the clinical variables, which included antipsychotic agent dosages of chlorpromazine equivalents, illness duration and PANSS scores. Age and gender effects were controlled, and multiple comparisons were corrected using the Bonferroni method (*P* < 0.05).

In addition, correlation analyses between rsFCD and clinical variables were performed in a voxel-wise manner in the whole brain. A linear regression model was used to perform correlation analyses with age and gender as covariates of no interest. Multiple comparisons were corrected using a FWE method (*P* < 0.05). To clarify the relationship between rsFCD and psychosis, we further divided the schizophrenia patients into patients with current psychotic symptoms (*n* = 57) and those without current psychotic symptoms (*n* = 38) and compared the rsFCD differences between the subgroups (*P* < 0.05, FWE correction).

## Results

### Subject demographics and clinical characteristics

The demographic and clinical characteristics of the subjects are summarized in Table 1. There were no significant group differences in sex (χ^2^ = 1.35, *P* = 0.25) or age (*t* = 0.48, *P* = 0.63). Eighty-seven patients received medications during the MRI examinations, and the remaining 8 patients had never received any medications. The mean dosage of antipsychotic agents (chlorpromazine equivalents) was 446.5 ± 341.6 mg/d for schizophrenia patients. The mean duration of illness was 121.4 ± 92.8 months. The mean scores of PANSS positive sub-scale, negative sub-scale and general psychopathology sub-scale were 17.1 ± 7.9, 20.3 ± 9.1, and 34.1 ± 10.8.

### rsFCD differences between schizophrenia patients and healthy controls

**Figure 1:**
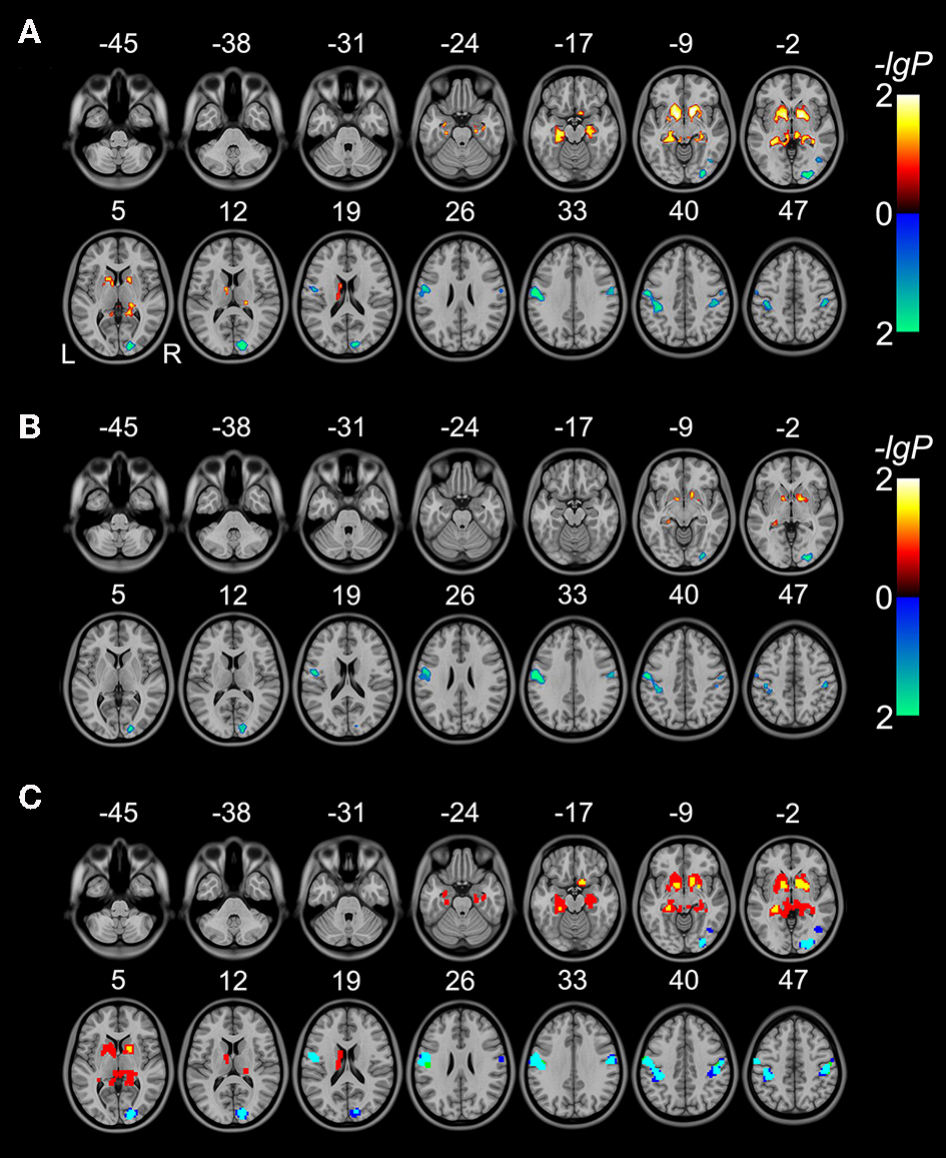
**Brain regions with significant differences in rsFCD (*P* < 0.05, FWE corrected) between schizophrenia patients and healthy comparison subjects without (A) and with (B) correction for GMV**. The warm color represents increased rsFCD and the cold color denotes decreased rsFCD in patients with schizophrenia. Overlap between significant rsFCD difference maps without and with GMV correction **(C)**. Red denotes increased rsFCD in schizophrenia without GMV correction; yellow represents increased rsFCD both without and with GMV correction. Blue and green denote decreased rsFCD without and with GMV correction, respectively; cyan represents decreased rsFCD both without and with GMV correction.

Without GMV correction, compared with HCs, schizophrenia patients exhibited increased rsFCD in the bilateral sub-cortical regions (including putamen, pallidum, left caudate body) and hippocampi and decreased rsFCD in the bilateral postcentral gyri, right inferior temporal gyrus and occipital cortex (Figure 1A and Table 2). After correction for GMV, the distribution of brain regions with significant differences in rsFCD was similar to that observed without GMV correction; however, the spatial extent was much smaller in the striatal and hippocampal regions (Figure 1B and Table 3). Significant rsFCD difference maps with and without GMV correction are overlapped in Figure 1C. In addition, brain regions with significant intergroup differences in rsFCD at the correlation thresholds of *R* > 0.2 and 0.4 are shown in Supplementary Figures S1, S2. Almost all of the significant brain regions at the threshold of *R* > 0.6 were included in the maps using thresholds of *R* > 0.2 and 0.4; however, the spatial extent of significant brain regions was much larger at the thresholds of *R* > 0.2 and 0.4 than that at the threshold of *R* > 0.6.

**Table 2:** **rsFCD changes in schizophrenia patients relative to healthy controls without GMV correction**.

**Brain regions**	**Brodmann areas**	**Cluster size (voxels)**	**Peak *t*-values**	**Coordinates in MNI (*x, y, z*)**
**SCHIZOPHRENIA PATIENTS > HEALTHY CONTROLS**				
Left putamen, pallidum	–	225	4.16	−12, 3, −6
Right putamen, pallidum	–	194	4.27	12, 9, −9
Left caudate body	–	38	2.99	−9,−3, 15
Left hippocampus	36, 35	270	3.81	−27, −36, −3
Right hippocampus	35	237	3.39	27, −30, −6
**SCHIZOPHRENIA PATIENTS < HEALTHY CONTROLS**				
Right occipital lobe	18	178	−4.80	27, −90, −3
Left postcentral gyrus	2, 3	241	−4.60	−57, −12, 33
Right postcentral gyrus	2, 3	111	−4.35	48, −24, 42
Right inferior temporal gyrus	37	25	−4.83	42, −66, −6

*Abbreviations: rsFCD, resting-state functional connectivity density; MNI, Montreal Neurological Institute*.

**Table 3:** **rsFCD changes in schizophrenia patients relative to healthy controls with GMV correction**.

**Brain regions**	**Brodmann areas**	**Cluster size (voxels)**	**Peak *t*-values**	**Coordinates in MNI (*x, y, z*)**
**SCHIZOPHRENIA PATIENTS > HEALTHY CONTROLS**				
Left putamen, pallidum	–	24	4.11	−12, 3, −6
Right putamen, pallidum	–	70	4.09	15, 3, −3
Left hippocampus	–	20	3.80	−27, −36, −3
**SCHIZOPHRENIA PATIENTS < HEALTHY CONTROLS**				
Right occipital lobe	18	107	−4.81	27, −90, −3
Left postcentral gyrus	2, 3	237	−4.67	−54, −12, 36
Right postcentral gyrus	2, 3	63	−4.01	48, −24, 45

*Abbreviations: rsFCD, resting-state functional connectivity density; MNI, Montreal Neurological Institute*.

### The association between rsFCD and clinical variables

In brain areas demonstrating group differences in rsFCD with correction for GMV, we did not find any statistical correlations between rsFCD and antipsychotic dosages of chlorpromazine equivalents, illness duration, and all PANSS scores (positive, negative, general psychopathology scores) (Supplementary Table S1). Additionally, no brain regions exhibited significant correlations between rsFCD and clinical variables in the voxel-based analyses (*P* < 0.05, FWE corrected). We did not observe any significant differences in rsFCD between patients with and without current psychotic symptoms (*P* < 0.05, FWE corrected).

## Discussion

In this study, we used a data-driven voxel-based method to compare rsFCD differences between schizophrenia patients and HCs. We found that schizophrenia patients exhibited increased rsFCD in the striatum and hippocampus and decreased rsFCD in the postcentral gyri and occipital cortex compared with HCs. The rsFCD changes in these regions reflect an alteration of total number of functional connections between these regions and all other voxels in the whole brain, representing an alteration of communication capacity and functional brain dynamics in these regions. Thus, our findings provide new evidence for the hypothesis that schizophrenia is a disorder of connectivity abnormalities from the perspective of functional connectivity density.

### Increased FCD in schizophrenia

In the present study, we found that schizophrenia patients had increased rsFCD in the striatum and hippocampus. The striatum and hippocampus are central components of the brain in the generation of psychosis and other symptom states in schizophrenia (Davis et al., 1991; Epstein et al., 1999; Howes and Kapur, 2009; Perez-Costas et al., 2010; Grace, 2012). Previous studies have demonstrated early striatal hypertrophy in first-episode psychosis (Chua et al., 2009) and significant correlations between GM density in the hippocampus and early psychotic symptoms in adolescents at risk of psychosis (Spencer et al., 2007). Several studies have suggested that the hyper-spontaneous neural activity in the striatum and hippocampus is a pathological feature of schizophrenia. For example, increased regional cerebral blood flow (rCBF) and amplitude of low frequency fluctuation (ALFF) in the striatum and hippocampus have been frequently reported in schizophrenia (Malaspina et al., 2004; Hoptman et al., 2010; Scheef et al., 2010; Pinkham et al., 2011; Liu et al., 2012; Turner et al., 2013). Moreover, previous studies have also reported increased rsFCS of the striatum and hippocampus with other parts of the brain (Salvador et al., 2010; Hoffman et al., 2011). However, Koch et al. found decreased striatal functional connectivity using psychophysiological interactions (PPI), which was closely linked to GMV changes of the striatum (Koch et al., 2014). Different methodologies (PPI vs. FCD) and experimental designs (task-evoked vs. resting-state) may account for the different findings between that study and ours. Although several previous studies suggested that increased functional activity and connectivity in these regions may be an effect of antipsychotic drugs, our non-significant correlations between aberrant rsFCD and antipsychotic dosages do not support this hypothesis. Our findings of increased rsFCD in the striatum and hippocampus may support the connectivity disorder hypothesis of schizophrenia from a new perspective of the number of functional connectivity.

After correcting for GMV, the spatial extent of the striatal and hippocampal regions with significant rsFCD differences between groups was much smaller than without GMV correction. This finding indicates that rsFCD increases in the striatal and hippocampal regions are partially related to GM atrophy. To clarify the specific relationship between rsFCD and GMV, we performed a correlation analysis in patients and controls, respectively. We did not find any significant correlations (Supplementary Table S2). These findings suggest that the relationship between GMV and rsFCD changes in schizophrenia is rather complex. In future studies on schizophrenia, the GMV effect on the functional changes (such as the rsFCD) should be considered.

### Decreased FCD in schizophrenia

We also found decreased rsFCD in the cortical regions involved in processing of sensorimotor and visual information; this effect is independent of GMV changes in these regions. These findings are consistent with a previous study that reported decreased local FCD in the sensorimotor cortex in schizophrenia (Tomasi and Volkow, 2014). The sensorimotor and visual areas also showed decreased rCBF (Pinkham et al., 2011), ALFF (Hoptman et al., 2010; Turner et al., 2013) and regional homogeneity (ReHo) (Liu et al., 2006; Yu et al., 2013) in schizophrenia. Hypoconnectivity of the visual and sensormotor networks has also been reported in schizophrenia (Collin et al., 2011; Tang et al., 2012; Calderone et al., 2013; Damaraju et al., 2014). Abnormal activity or connectivity of the visual cortex may be associated with deficits in visual processing and object-recognition in schizophrenia (Onitsuka et al., 2007; Wynn et al., 2008). Similarly, abnormal activity or connectivity of the sensorimotor cortex may be related to neurological soft signs that cause neurological abnormalities in sensory integration, motor regulation, and sequencing complex motor acts in schizophrenia patients (Schmauss et al., 1993). Combined with these previous studies, our findings further support the functional disconnectivity hypothesis of schizophrenia from the perspective of connectivity density.

### Association between FCD changes and the dopamine hypothesis of schizophrenia

A previous imaging genetics study revealed that FCD can be modulated by the presumed dopamine signaling in healthy subjects, and the modulation pattern is different in different regions (Tian et al., 2013). This study found an association between FCD and dopamine activity. According to the dopamine hypothesis of schizophrenia, hyperdopaninergia in the striatum, and hypodopaminergia in the cortical regions, especially the prefrontal cortex, have been conceived as an important pathological feature of schizophrenia (Howes and Kapur, 2009). Dopamine neuron responsivity is shown to be modulated by the hippocampus, which exhibits hyperactivity in schizophrenia (Grace, 2012). Therefore, hyperdopaninergia in the striatum may reflect an overly responsive state of the dopamine system that is driven by hyperactivity in the hippocampus. This theoretical hypothesis may explain our findings of increased rsFCD in the striatum and hippocampus in schizophrenia. In contrast to the dominant dopamine receptor D2 in the striatum, the dominant dopamine receptor is D1 in the cerebral cortex. D1 dysfunction in the prefrontal cortex has been linked to cognitive impairment and negative symptoms in schizophrenia (Abi-Dargham and Moore, 2003; Goldman-Rakic et al., 2004; Laruelle, 2014). The distribution differences in dopamine receptors between the striatum and cortex may be an explanation for the completely different rsFCD changes in these two kinds of brain regions in schizophrenia. However, most previous studies focused on dopamine activity in the prefrontal cortex; whether the influence of this activity can be applied to the sensory and motor areas need to be further determined. However, there is a lack of direct evidence for an association between FCD changes and dopamine activity; thus, future studies are needed to clarify this potential association.

In this present study, we did not find a correlation between aberrant rsFCD and antipsychotic dose, illness duration, and symptom severity. These results suggest that aberrant rsFCD may be an independent trait characteristic of schizophrenia, which means that all schizophrenia patients have aberrant rsFCD, regardless of symptom severity. This is not surprising, given that many studies have shown that illness duration, symptom severity, and antipsychotic dose are not linearly related to brain functional activity disturbances. Furthermore, we did not observe any differences in rsFCD between patients with and without current psychotic symptoms. BOLD signal alterations may better track schizophrenia, regardless of the presence or absence of psychosis, rather than psychosis severity or duration and are not linearly related to antipsychotic dose (Woodward et al., 2012; Ren et al., 2013). However, we cannot rule out the possibility that either a lack of statistical power or a lack of data assessing the appropriate dependent clinical variables may account for non-significant correlations between rsFCD and demographic and clinical variables in our study. Therefore, in future studies, appropriate statistical methods and detailed clinical variables may contribute to the solution of this problem.

## Conclusion

Our study found that increased rsFCD in the striatal and hippocampal regions and decreased rsFCD in the sensorimotor regions are pathological features of schizophrenia, which may support the hypothesis that schizophrenia is a connectivity disorder from the perspective of functional connectivity density. The similar spatial distribution and abnormal patterns between brain regions with rsFCD alterations and those with dopamine alterations are interesting findings and deserve further investigations.

### Conflict of interest statement

The authors declare that the research was conducted in the absence of any commercial or financial relationships that could be construed as a potential conflict of interest.
