# Supplementary material for: Functional connectivity density alterations in schizophrenia
Source: Front Behav Neurosci. 2014 Nov 19;8:404. doi: 10.3389/fnbeh.2014.00404 (PMC4237131; doi:10.3389/fnbeh.2014.00404)
Supplement: Supplementary file 1 [file Image1.PDF]

Supplementary Materials

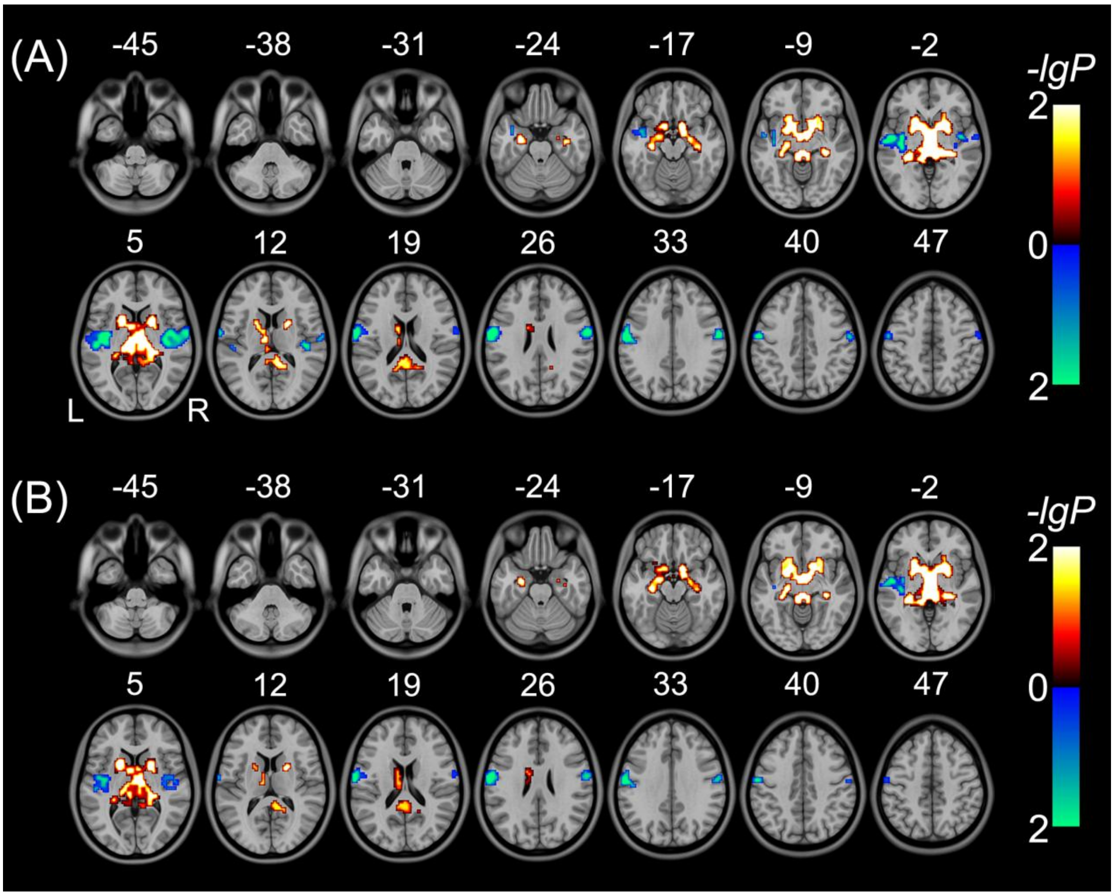

Figure S1. Brain regions with significant differences in rsFCD ($P < 0.05$, FWE corrected) at the threshold of $R > 0.2$ between schizophrenia patients and healthy comparison subjects without (A) and with (B) correction for GMV. The warm color represents increased rsFCD and the cold color denotes decreased rsFCD in patients with schizophrenia.

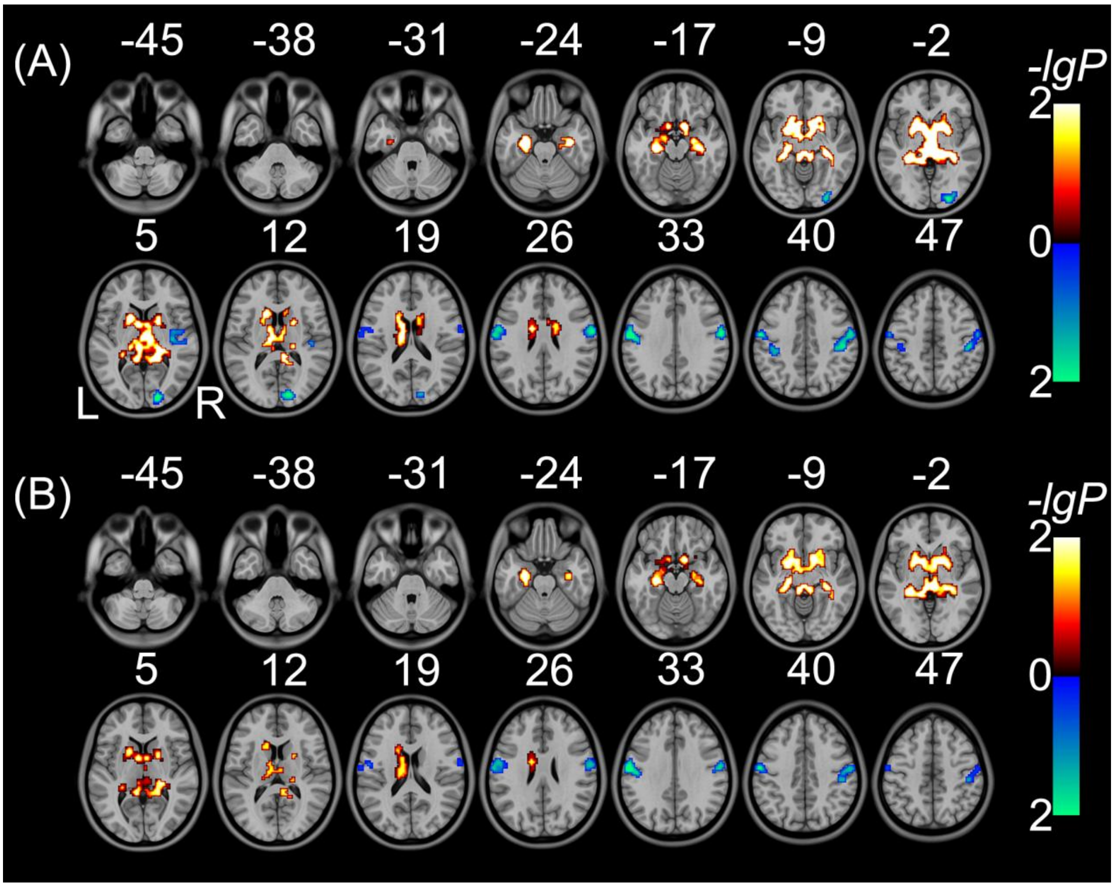

Figure S2. Brain regions with significant differences in rsFCD (*P* < 0.05, FWE corrected) at the threshold of R > 0.4 between schizophrenia patients and healthy comparison subjects without (A) and with (B) correction for GMV. The warm color represents increased rsFCD and the cold color denotes decreased rsFCD in patients with schizophrenia.

Table S1. Correlation Analyses between rsFCD and Clinical Variables in Schizophrenia Patients

Brain Regions	Antipsychotic dosages	Illness duration	Positive score	Negative score	General score
Schizophrenia patients > Healthy controls					
Left putamen, pallidum	0.09(0.40)	-0.01(0.93)	-0.11(0.31)	0.20(0.06)	<0.01(0.99)
Right putamen, pallidum	0.18(0.08)	-0.20(0.05)	-0.02(0.88)	<0.01(0.97)	-0.08(0.43)
Left hippocampus	0.04(0.72)	0.08(0.44)	-0.05(0.66)	0.12(0.27)	-0.05(0.64)
Schizophrenia patients < Healthy controls					
Right occipital lobe	-0.07(0.51)	-0.14(0.17)	0.06(0.55)	0.13(0.23)	0.08(0.43)
Left postcentral gyrus	-0.07(0.52)	-0.16(0.13)	0.02(0.85)	<0.01(0.99)	-0.03(0.74)
Right postcentral gyrus	0.02(0.89)	-0.21(0.05)	-0.06(0.56)	-0.09(0.40)	-0.07(0.54)

Data are shown as the *pr* value (*P* value). The *P* values are uncorrected and *pr* denotes partial correlation coefficient.

Table S2. Correlation Analyses between rsFCD and GMV in Brain Regions with Significant Group Differences in rsFCD without Correction for GMV

Brain Regions	Schizophrenia Patients	Healthy Subjects
Schizophrenia patients > Healthy controls		
Left putamen, pallidum	-0.12(0.25)	0.06(0.54)
Right putamen, pallidum	0.01(0.96)	0.12(0.25)
Left caudate body	-0.08(0.43)	0.10(0.35)
Left hippocampus	0.11(0.28)	-0.04(0.73)
Right hippocampus	-0.01(0.94)	-0.03(0.78)

Data are shown as the *r* value (*P* value). The *P* values are uncorrected and *r* denotes Pearson correlation coefficient.
